# LDL‐C Estimation in Ghana: A Comparative Study of 13 Calculation Methods

**DOI:** 10.1155/bmri/8217938

**Published:** 2026-05-28

**Authors:** Samuel Asamoah Sakyi, Linda Ahenkorah Fondjo, Raphael Osei Mensah-Bonsu, Oscar Simon Olympio Mensah, Samuel Kwarteng, Samuel Kwame Sopuruchi Agomuo, Edward Osei Gyimah, Harriet Yeboaa Diawuo, Godfred Yawson Scott, Richmond Gorman, Beatrice Nyantakyiwaa, George Oduro Owusu, Ebenezer Senu, Stephen Opoku, Alfred Effah, Benjamin Amoani

**Affiliations:** ^1^ Department of Molecular Medicine, School of Medical Sciences, Kwame Nkrumah University of Science and Technology, Kumasi, Ghana, knust.edu.gh; ^2^ Department of Medical Diagnostics, Faculty of Allied Health Sciences, Kwame Nkrumah University of Science and Technology, Kumasi, Ghana, knust.edu.gh; ^3^ Department of Clinical Microbiology, School of Medical Sciences, Kwame Nkrumah University of Science and Technology, Kumasi, Ghana, knust.edu.gh; ^4^ Faculty of Pharmacy and Pharmaceutical Sciences, Kwame Nkrumah University of Science and Technology, Kumasi, Ghana, knust.edu.gh; ^5^ Department of Biological Sciences, School of Natural Sciences and Mathematics, The University of Texas at Dallas, Richardson, Texas, USA, utdallas.edu; ^6^ Division of Clinical Immunology and Rheumatology, University of Alabama at Birmingham, Birmingham, Alabama, USA, uab.edu; ^7^ Department of Biomedical Science, School of Allied Health Sciences, University of Cape Coast, Cape Coast, Central Region, Ghana, ucc.edu.gh

**Keywords:** Chen and Zhang formula, Friedewald formula, LDL cholesterol

## Abstract

**Background:**

Friedewald′s equation remains the most widely used method for estimating LDL‐C in clinical settings, despite the availability of several homogeneous direct assays. However, its accuracy declines under certain clinical conditions, such as unusually high or low levels of triglycerides and total cholesterol. Recently, several novel population‐specific equations have been reported to outperform the Friedewald equation. This study therefore is aimed at evaluating and comparing the accuracy of 13 LDL‐C estimation equations within the Ghanaian population.

**Methods:**

A cross‐sectional study was conducted among 976 individuals at Suntreso Government Hospital, Kumasi, Ghana. Fasting venous blood samples were analyzed for lipid profiles. Direct LDL‐C was enzymatically measured and compared with values calculated using 13 equations. Performance was assessed with Pearson correlations, Bland–Altman plots, and Wilcoxon signed‐rank tests.

**Results:**

All 13 equations correlated significantly with direct LDL‐C (*r* = 0.563–0.857, *p* < 0.001), with DeLong, Puavilai, Vujovic, Sampson, Choi, and Martin showing the strongest correlations (*r* = 0.857). Ahmadi (71.39 mg/dL), Hattori (44.93 mg/dL), Rao (25.8 mg/dL), and Choi (19.58 mg/dL) were the most overestimated LDL‐C formulas, while de Cordova (−5.59 mg/dL) underestimated the direct LDL‐C measurement. Subgroup analyses by age, triglyceride, and total cholesterol levels revealed that the Friedewald and Chen and Zhang formulas exhibited nonsignificant median differences (*p* > 0.05) in individuals aged 18–55 years and those with triglyceride levels ≥ 200 mg/dL.

**Conclusion:**

The Sampson, Martin, and Puavilai equations demonstrated the most reliable and consistent performance across age and lipid subgroups and should be considered preferred methods for LDL‐C estimation in clinical and research settings in Ghana. Although Friedewald and Chen and Zhang performed adequately in younger adults and individuals with elevated triglycerides, their overall agreement was weaker, highlighting the broader utility of the Sampson, Martin, and Puavilai equations.

## 1. Introduction

Cardiovascular diseases (CVDs) are the leading cause of death globally, resulting in an estimated 17.9 million deaths annually [1]. CVDs encompass a range of disorders affecting the heart and blood vessels, including coronary heart disease (CHD) [1]. Increased low‐density lipoprotein cholesterol (LDL‐C) can be described as a strong predictor of CHD, significantly associated with atherosclerosis [2]. The Adult Treatment Panel III (ATP III) of the National Cholesterol Education Program (NCEP) highlights LDL‐C concentration as the primary factor for treatment decisions and patient risk stratification, emphasizing the need for accurate and precise LDL‐C analysis [3].

While beta‐quantification is the gold standard for LDL‐C measurement, its complexity limits its use in routine labs [4, 5]. Alternative methods involve the direct measurement of LDL‐C using homogeneous assays, which tend to perform poorly in the presence of elevated triglyceride (TG) [6, 7]. These techniques are costly, less convenient, and often unavailable in routine laboratory settings [5]. Ultracentrifugation, while a precise separation method, is time‐consuming, and its high salt concentrations and centrifugal forces can significantly alter fragile lipoproteins [8].

Friedewald′s formula remains the most widely used method for calculating LDL‐C despite limitations in accuracy at extreme TG or total cholesterol (TC) levels [9] or in patients with comorbidities [9, 10]. While alternative formulas have been proposed [11], they have inconsistently outperformed Friedewald′s formula (Christeen [12]), showing varying results across different populations [11, 12]. A previous large‐scale study involving 23,055 patients found no significant advantage of a novel formula over Friedewald′s formula, showing a positive bias at low LDL‐C levels (< 1.81 mmol/L) [13]. Although de Cordova et al.′s formula outperformed Friedwald′s formula across a wide range of lipid levels in a Brazilian study involving 10,664 patients [14], subsequent research in a South African cohort of 576 healthy subjects with the same formula showed bias at low LDL‐C levels [15].

The development of more accurate and reliable methods for calculating LDL‐C has become increasingly essential due to the rising prevalence of CVDs [16, 17], especially among adults in Ghana [18–20]. The paucity of data on modified LDL‐C formulas in the Ghanaian population makes it necessary for existing formulas to be documented and validated. As treatment decisions such as statin initiation and cardiovascular risk stratification rely heavily on LDL‐C estimation [21], identifying an optimal formula could have a significant clinical impact in Ghanaian healthcare settings. It is against this background that we evaluated the applicability of 13 LDL‐C calculation formulas using a large sample size in the Ghanaian population.

## 2. Materials and Methods

### 2.1. Study Design and Site

This hospital‐based cross‐sectional study was conducted at the Suntreso Government Hospital, located in Kumasi, the Regional Capital of the Ashanti Region of Ghana. This hospital takes direct referrals from major towns and cities in the Ashanti Region, as well as from 12 out of the 16 administrative regions in Ghana.

### 2.2. Study Population

A total of 976 individuals who visited the Suntreso Government Hospital laboratory, 18 years and above, were recruited for this study. Participants with no evidence of metabolic conditions including diabetes, renal dysfunction, cholestatic liver disease, and hypo‐ or hyperthyroidism as per clinical history were included. Additionally, participants who observed at least 10 h of overnight fasting were recruited for participation.

### 2.3. Ethical Considerations

Ethical approval was sought from the Committee on Human Research, Publication and Ethics (CHRPE) of the School of Medical Sciences, KNUST (CHRPE/AP/1331/24), and the ethics committee of the Suntreso Government Hospital. All eligible participants provided written informed consent after being fully informed about the study′s purpose, procedures, potential risks, and benefits, in accordance with the Declaration of Helsinki. The confidentiality of the data collected was assured, and all data were handled anonymously.

### 2.4. Sample Collection and Processing

Five milliliters of venous blood samples was collected after an overnight fast and transferred into plain blood tubes. Blood samples were then centrifuged at 3000 rpm for 15 min after an hour of coagulation. All samples were analyzed for lipid profile parameters, including high‐density lipoprotein cholesterol (HDL‐C), LDL‐C, TG, and TC.

### 2.5. Lipid Profile Analysis

Lipid profiles were quantified using a standard homogeneous enzymatic colorimetric assay with an automated chemistry analyzer (Hitachi 902, Roche Germany). Calibration and internal quality controls (QCs) were provided by Pars Azmoon Company, with calibration traceability ensured through Cholesterol Reference Method Laboratory Network (CRMLN) standards [22]. Internal QC sera at three concentration levels were analyzed to monitor assay precision, with the coefficients of variation (CVs) consistently ≤ 4%, in accordance with Roche manufacturer specifications and international performance standards.TC was measured enzymatically through coupled reactions involving cholesteryl ester hydrolase, cholesterol oxidase, and peroxidase, producing hydrogen peroxide (H_2_O_2_), which was quantified colorimetrically at 500 nm. TGs were hydrolyzed to glycerol, oxidized by glycerol oxidase to produce H_2_O_2_, and measured similarly. HDL‐C was determined directly by treating samples with a blocking reagent to remove Apolipoprotein B–containing lipoproteins, followed by enzymatic reaction with sulfated alpha‐cyclodextrin, polyethylene glycol–coupled cholesteryl esterase, and cholesterol oxidase. LDL‐C was measured directly by separating LDL from chylomicrons, very low‐density lipoprotein (VLDL), and HDL‐C, followed by an enzymatic colorimetric assay.

LDL‐C was calculated for each participant using the following 13 equations, with TC, HDL‐C, and TG in milligrams per deciliter:

1. Friedewald: LDL‐C = TC – HDL‐C – (TG/5) [23].
2. Martin/Hopkins: LDL‐C = TC – HDL‐C – (TG/adjustable factor) [24].
3. Sampson: LDL‐C = (TC/0.948) – (HDL‐C/0.971) – (TG/8.56 + TG × non‐HDL‐C/2140 – TG^²^/16, 100) – 9.44 [25].
4. DeLong: LDL‐C = TC – (HDL‐C + 0.16 × TG) [26].
5. Rao: LDL‐C = (4.7 × TC – 4.364 × HDL‐C – TG)/4.487 [27]
6. Choi: LDL‐C = TC – (0.87 × HDL‐C) – (0.13 × TG) [28].
7. Chen: LDL‐C = (TC – HDL‐C) × 0.9 – (TG × 0.1) [29].
8. de Cordova: LDL‐C = 0.7516 × (TC – HDL‐C) [14].
9. Vujovic: LDL‐C = TC – HDL‐C – (TG/6.85) [30].
10. Anandaraja: LDL‐C = (0.9 × TC) – (0.9 × TG/5) – 28 [31].
11. Hattori: LDL‐C = (0.94 × TC) – (0.94 × HDL‐C) – (0.19 × TG) [32].
12. Ahmadi: LDL‐C = (TC/1.19) + (TG/1.9) – (HDL‐C/1.1) – 38 [33].
13. Puavilai: LDL‐C = TC – HDL‐C – (TG/6) [34].

### 2.6. Statistical Analysis

Statistical analyses were performed using Statistical Package for Social Sciences (SPSS Version 26.0) and R programming language (Version 4.4.4). The distribution of the continuous variable was determined by the Kolmogorov–Smirnov test for normality. Continuous variables were presented as means with standard deviations for parametric variables or medians with interquartile ranges (IQRs) for nonparametric variables. Comparison of continuous variables between independent groups (male and female) was performed using the independent samples *t*‐test for normally distributed variables or the Mann–Whitney *U* test for nonparametric variables. Pearson correlation coefficients (*r*) were calculated to assess the linear relationship between estimated and directly measured LDL‐C for each equation. Bland–Altman plots were generated to evaluate agreement between estimated and directly measured LDL‐C, quantifying mean bias and limits of agreement. Data were then stratified by age, TG, and TC levels to assess subgroup‐specific performance of all 13 equations. For each subgroup, median LDL‐C values and IQRs were computed for direct and estimated LDL‐C. Median differences were calculated to assess bias. To evaluate the clinical relevance of each LDL‐C estimation equation, a categorical agreement analysis was performed. Each patient′s directly measured and estimated LDL‐C values were independently assigned to one of eight guideline‐based clinical categories: < 40, 40–54, 55–69, 70–99, 100–129, 130–159, 160–189, and ≥ 190 mg/dL [35]. For each equation, the proportion of patients assigned to the same category as the direct LDL‐C measurement was calculated and expressed as a percentage, both within each category and overall. The Wilcoxon signed‐rank test was used to determine the statistical significance of differences between estimated and direct LDL‐C medians. Correlation coefficients (*r*) were also computed for each subgroup to evaluate equation performance across demographic and clinical characteristics. *p* values less than 0.05 were considered statistically significant.

## 3. Results

### 3.1. Demographic and Biochemical Characteristics of Study Participants

Of the 976 study participants, the majority, 765 (78.4%), were females, while the remaining were males, 211 (21.6%). The mean age of participants was 51.61 ± 15.58 years, with no significant difference between females and males (*p* = 0.414). Females had significantly higher median levels of TC (184.86 vs. 178.00 mg/dL, *p* = 0.019) and HDL‐C (47.58 vs. 44.00 mg/dL, *p* = 0.009) compared to males. Among the various LDL‐C estimation methods, significant gender differences were observed in values derived from the Anandaraja (*p* = 0.021), Vujovic (*p* = 0.049), Chen and Zhang (*p* = 0.048), Choi (*p* = 0.041), Martin (*p* = 0.049), and Sampson (*p* = 0.047) formulas, with females generally exhibiting higher LDL‐C levels than males. Other lipid parameters, including TGs, VLDL, direct LDL‐C, and most alternative LDL‐C estimations, showed no statistically significant differences between genders (*p* > 0.05) (Table 1).

**Table 1:** Demographic and biochemical characteristics stratified by gender.

Parameter	Total (*n* = 976)	Female (*n* = 765)	Male (*n* = 211)	*p* value
Age (years)	51.61 ± 15.58	51.82 ± 15.45	50.83 ± 16.06	0.414
Total cholesterol (mg/dL)	182.96 (151.32–223.00)	184.86 (154.44–226.10)	178.00 (142.00–213.33)	**0.019**
Triglycerides (mg/dL)	109.00 (85.00–150.85)	108.00 (85.22–150.00)	112.00 (83.66–151.30)	0.898
HDL‐C (mg/dL)	47.00 (35.49–62.01)	47.58 (36.27–63.18)	44.00 (34.71–56.16)	**0.009**
Non‐HDL‐C (mg/dL)	134.94 (105.30–170.82)	136.50 (107.00–172.00)	128.31 (99.84–159.12)	0.051
VLDL (mg/dL)	21.80 (17.00–30.17)	21.60 (17.04–30.00)	22.40 (16.73–30.26)	0.896
Direct LDL‐C	104.33 (79.00–136.88)	105.00 (80.20–137.67)	100.62 (75.66–132.60)	0.193
Friedewald LDL‐C	111.56 (82.83–143.40)	112.44 (84.44–145.25)	106.60 (76.74–137.80)	0.057
DeLong LDL‐C	116.73 (87.56–148.18)	117.24 (88.80–150.63)	109.37 (81.40–144.13)	0.051
Rao LDL‐C	128.73 (98.46–165.06)	129.78 (100.30–167.85)	122.98 (93.02–155.62)	0.055
Hattori LDL‐C	148.17 (120.38–186.33)	148.73 (121.82–187.40)	143.23 (113.97–176.86)	**0.070**
Anandaraja LDL‐C	116.11 (86.30–149.78)	117.26 (88.82–152.52)	110.75 (78.57–144.36)	**0.021**
Ahmadi LDL‐C	169.51 (129.52–218.17)	169.18 (130.53–220.53)	169.67 (123.68–214.01)	0.356
Puavilai LDL‐C	115.65 (86.82–147.45)	116.41 (88.02–149.73)	108.96 (80.50–142.74)	0.052
Vujovic LDL‐C	117.58 (88.27–148.96)	118.48 (89.99–151.74)	109.87 (82.48–145.80)	**0.049**
Chen and Zhang LDL‐C	109.41 (83.67–138.91)	110.44 (85.25–140.75)	104.21 (78.70–134.20)	**0.048**
de Cordova LDL‐C	101.42 (79.14–128.39)	102.59 (80.42–129.27)	96.44 (75.04–119.59)	0.051
Choi LDL‐C	126.48 (96.38–158.76)	127.08 (99.39–161.16)	119.82 (90.18–153.64)	**0.041**
Martin LDL‐C	113.49 (84.78–143.62)	114.13 (86.56–145.77)	106.06 (79.72–140.29)	**0.049**
Sampson LDL‐C	113.89 (84.72–145.87)	114.85 (86.61–148.36)	108.05 (78.98–141.54)	**0.047**

*Note:* Data presented as mean ± standard deviation or median (interquartile range) depending on the normality. Independent samples *t*‐test or Mann–Whitney *U* test *p* values were presented depending on the normality (*p* < 0.05), and values in bold were considered statistically significant.

Abbreviations: HDL‐C, high‐density lipoprotein cholesterol; LDL‐C, low‐density lipoprotein cholesterol; VLDL, very low‐density lipoprotein.

### 3.2. Correlation Between Estimated LDL‐C by 13 Formulas and Directly Measured LDL‐C

**Figure 1:**
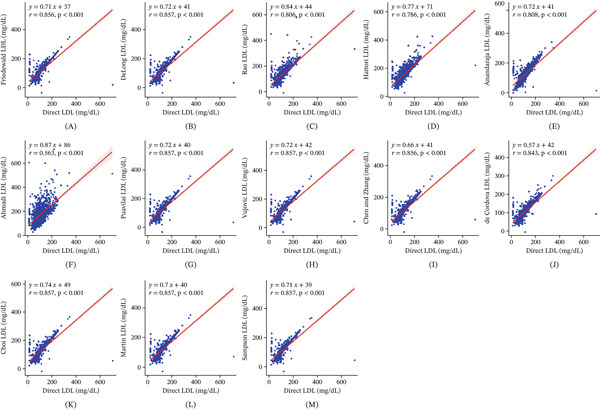
Correlation of calculated LDL by (A) Friedewald, (B) DeLong, (C) Rao, (D) Hattori, (E) Anandaraja, (F) Ahmadi, (G) Puavilai, (H) Vujovic, (I) Chen and Zhang, (J) de Cordova, (K) Choi, (L) Martin, and (M) Sampson formulas with directly measured LDL.

Figure 1A–M presents scatter plots illustrating the correlation between directly measured LDL‐C and LDL‐C values estimated by 13 different equations. All equations showed statistically significant positive correlations (*p* < 0.001), with correlation coefficients (*r*) ranging from 0.563 to 0.857. The DeLong, Puavilai, Vujovic, Sampson, Choi, and Martin equations demonstrated the strongest correlations (*r* = 0.857), followed by Friedewald and Chen and Zheng (*r* = 0.856). Other equations, including de Cordova (*r* = 0.843), Anandaraja (*r* = 0.808), Rao (*r* = 0.806), and Hattori (*r* = 0.786), exhibited strong correlations. The Ahmadi equation displayed the weakest correlation among the 13 formulas (*r* = 0.563).

### 3.3. Agreement Between Directly Measured LDL‐C and Calculated LDL‐C

**Figure 2:**
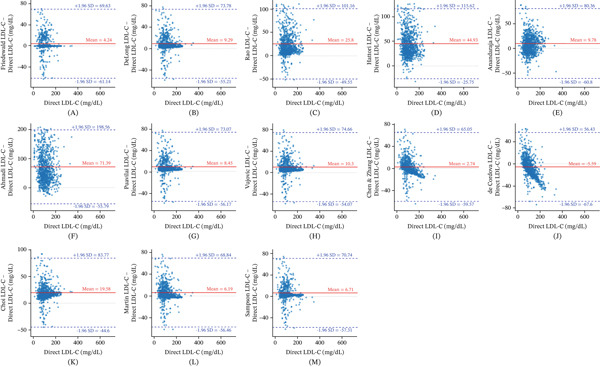
Bland–Altman plots of direct LDL‐C and (A) Friedewald, (B) DeLong, (C) Rao, (D) Hattori, (E) Anandaraja, (F) Ahmadi, (G) Puavilai, (H) Vujovic, (I) Chen and Zhang, (J) de Cordova, (K) Choi, (L) Martin, and (M) Sampson calculated LDL‐C.

Figure 2 shows Bland–Altman plots comparing directly measured LDL‐C with LDL‐C levels estimated using 13 different formulas, assessing agreement and bias. Most formulas displayed acceptable limits of agreement; however, notable differences in mean bias were observed. The Rao (25.8 mg/dL), Ahmadi (71.39 mg/dL), Hattori (44.93 mg/dL), and Choi (19.58 mg/dL) formulas significantly overestimated LDL‐C compared to the direct measurement, while the DeLong (mean bias: 9.29 mg/dL), Anandaraja (9.78 mg/dL), Vujovic (10.3 mg/dL), Puavilai (8.45 mg/dL), Martin (6.19 mg/dL), Sampson (6.71 mg/dL), Friedewald (4.24 mg/dL), and Chen and Zhang (2.74 mg/dL) equations slightly overestimated LDL‐C. The de Cordova equation (−5.59 mg/dL) underestimated LDL‐C compared to the direct measurement.

### 3.4. Categorical Agreement for Risk Stratification Using Other LDL‐C Calculations

Table 2 presents the categorical agreement of the 13 estimation equations relative to dLDL‐C measurement. The Friedewald equation demonstrated the highest overall classification accuracy (79.9%), followed by Sampson (73.6%), Martin (71.1%), Chen and Zhang and Puavilai (both 68.9%), and DeLong (66.5%). Vujovic (63.4%), de Cordova (52.7%), Anandaraja (53.0%), Rao (41.8%), and Choi (40.9%) showed intermediate performance. The poorest overall agreement was recorded for Hattori (16.9%) and Ahmadi (17.1%), with fewer than one in five patients correctly classified by either equation.

**Table 2:** Categorical agreement for risk stratification using other LDL‐C calculations.

Equation (direct LDL‐C)	LDL‐C category (mg/dL)								Overall (*n* = 976)
	< 40 (*n* = 35)	40–54 (*n* = 39)	55–69 (*n* = 89)	70–99 (*n* = 286)	100–129 (*n* = 235)	130–159 (*n* = 155)	160–189 (*n* = 77)	≥ 190 (*n* = 60)	
Ahmadi	0.00	0.00	6.70	12.60	15.30	14.20	3.90	97.00	17.10
Anandaraja	28.60	35.90	32.60	54.50	54.00	54.80	45.50	97.00	53.00
Chen and Zhang	5.70	35.90	61.80	72.70	75.30	71.60	72.70	80.60	68.90
Choi	5.70	5.10	12.40	40.20	40.00	49.70	45.50	98.50	40.90
de Cordova	5.70	35.90	58.40	74.10	56.60	34.80	24.70	47.80	52.70
DeLong	14.30	35.90	50.60	63.60	68.90	78.10	76.60	98.50	66.50
Friedewald	28.60	69.20	75.30	75.20	81.30	91.00	88.30	98.50	79.90
Hattori	0.00	0.00	5.60	11.90	13.20	16.10	5.20	100.00	16.90
Martin	8.60	41.00	52.80	69.90	75.30	79.40	89.60	95.50	71.10
Puavilai	14.30	41.00	53.90	67.10	71.50	78.70	77.90	98.50	68.90
Rao	5.70	10.30	11.20	42.70	45.10	45.80	37.70	100.00	41.80
Sampson	11.40	48.70	65.20	70.30	76.20	85.20	83.10	98.50	73.60
Vujovic	8.60	28.20	40.40	63.30	64.30	76.80	72.70	98.50	63.40

*Note:* Data presented as percentages (%).

Abbreviations: LDL‐C, low‐density lipoprotein cholesterol; mg/dL, milligrams per deciliter.

### 3.5. Estimation of LDL‐C in Four Subgroups Based on Age Ranges

Table 3 presents the median (IQR) values of LDL‐C calculated using 13 different formulas across various age groups and compares them with directly measured LDL‐C values in terms of median differences, correlation coefficients, and Wilcoxon signed‐rank test *p* values. Across all age ranges, the Sampson, Martin, Puavilai, and Friedewald formulas consistently showed the smallest median differences from direct LDL‐C and high correlation coefficients (*r* ≥ 0.86), indicating strong agreement. The Ahmadi formula exhibited the largest positive bias, with median differences ranging from 49.79 to 62.66 mg/dL, and lower correlations (*r* = 0.41–0.79), suggesting it significantly overestimated LDL‐C across all age groups. Conversely, the de Cordova formula tended to underestimate LDL‐C, particularly in older adults (≥ 56 years), though it still maintained moderate to high correlations. The Rao and Hattori formulas also showed consistent overestimations with moderate correlations. Among younger adults (18–35 years), the Chen and Zhang and Friedewald formulas had negligible median differences and nonsignificant *p* values, reflecting good agreement. While all formulas demonstrated statistically significant correlations with direct LDL‐C (except Friedewald and Chen and Zhang in younger adults), the Sampson, Martin, and Puavilai equations emerged as the most accurate and consistent across all age categories.

**Table 3:** Median (IQR) values of calculated LDL‐C by 13 formulas for various age ranges and their correlation with directly measured LDL‐C.

Method	Median (IQR)	Median difference (mg/dL)	Correlation (*r*)	Wilcoxon *p*
Age 18–35 years (*n* = 183)				
Direct LDL‐C	93.60 (73.32–116.00)	—	—	—
Friedewald LDL‐C	91.76 (72.33–115.68)	0.02	0.966	0.444
DeLong LDL‐C	96.26 (77.60–120.08)	4.28	0.963	**< 0.001**
Rao LDL‐C	108.10 (88.69–140.79)	11.87	0.797	**< 0.001**
Hattori LDL‐C	128.22 (108.22–160.83)	33.69	0.750	**< 0.001**
Anandaraja LDL‐C	89.38 (66.23–114.02)	−5.24	0.906	**0.001**
Ahmadi LDL‐C	145.62 (113.03–205.87)	49.79	0.410	**< 0.001**
Puavilai LDL‐C	95.30 (77.09–119.31)	3.65	0.964	**< 0.001**
Vujovic LDL‐C	97.18 (78.20–121.00)	5.10	0.962	**< 0.001**
Chen and Zhang LDL‐C	91.11 (75.33–115.26)	−1.29	0.956	0.138
de Cordova LDL‐C	86.18 (70.64–107.87)	−7.61	0.906	**< 0.001**
Choi LDL‐C	104.78 (85.13–130.14)	13.43	0.960	**< 0.001**
Martin LDL‐C	93.33 (75.84–118.14)	0.56	0.960	**0.002**
Sampson LDL‐C	93.82 (74.97–117.44)	1.90	0.964	**< 0.001**
Age 36–55 years (*n* = 394)				
Direct LDL‐C	107.00 (81.18–136.50)	—	—	—
Friedewald LDL‐C	111.30 (82.92–139.30)	0.00	0.866	0.173
DeLong LDL‐C	115.48 (87.96–144.57)	5.14	0.867	**< 0.001**
Rao LDL‐C	126.79 (101.07–165.26)	17.02	0.810	**< 0.001**
Hattori LDL‐C	146.30 (122.25–185.26)	39.18	0.786	**< 0.001**
Anandaraja LDL‐C	116.60 (89.69–144.76)	5.21	0.802	**< 0.001**
Ahmadi LDL‐C	170.03 (132.93–216.11)	62.66	0.519	**< 0.001**
Puavilai LDL‐C	114.75 (87.07–143.76)	4.30	0.867	**< 0.001**
Vujovic LDL‐C	116.16 (88.94–145.37)	6.11	0.867	**< 0.001**
Chen and Zhang LDL‐C	108.15 (83.96–135.35)	‐0.70	0.866	0.825
de Cordova LDL‐C	99.52 (80.39–127.14)	‐8.27	0.851	**< 0.001**
Choi LDL‐C	124.88 (96.41–154.75)	15.99	0.867	**< 0.001**
Martin LDL‐C	111.53 (85.31–140.58)	1.74	0.867	**< 0.001**
Sampson LDL‐C	113.20 (85.35–141.90)	2.80	0.867	**< 0.001**
Age 56–79 years (*n* = 355)				
Direct LDL‐C	113.00 (80.00–149.37)	—	—	—
Friedewald LDL‐C	123.60 (91.00–157.17)	0.00	0.824	**< 0.001**
DeLong LDL‐C	128.40 (95.80–162.09)	5.40	0.824	**< 0.001**
Rao LDL‐C	140.20 (106.91–180.60)	17.07	0.804	**< 0.001**
Hattori LDL‐C	161.06 (128.19–200.12)	37.14	0.789	**< 0.001**
Anandaraja LDL‐C	131.30 (97.42–169.24)	12.24	0.805	**< 0.001**
Ahmadi LDL‐C	177.67 (131.93–229.67)	52.48	0.644	**< 0.001**
Puavilai LDL‐C	127.83 (95.00–161.27)	4.50	0.824	**< 0.001**
Vujovic LDL‐C	129.24 (96.76–163.07)	6.48	0.824	**< 0.001**
Chen and Zhang LDL‐C	121.40 (92.30–152.10)	‐1.27	0.824	0.3745
de Cordova LDL‐C	110.51 (86.18–139.80)	‐10.53	0.816	**< 0.001**
Choi LDL‐C	139.02 (105.80–173.00)	17.84	0.827	**< 0.001**
Martin LDL‐C	124.81 (94.29–157.18)	1.40	0.824	**< 0.001**
Sampson LDL‐C	126.38 (93.42–160.00)	3.01	0.825	**< 0.001**
Age ≥ 80 years (*n* = 44)				
Direct LDL‐C	110.50 (81.75–131.75)	—	—	—
Friedewald LDL‐C	116.15 (89.16–145.41)	0.07	0.886	**0.006**
DeLong LDL‐C	121.38 (92.72–150.13)	6.04	0.896	**< 0.001**
Rao LDL‐C	138.90 (96.40–163.20)	19.85	0.892	**< 0.001**
Hattori LDL‐C	159.45 (121.51–186.77)	42.18	0.876	**< 0.001**
Anandaraja LDL‐C	119.60 (99.80–152.75)	13.78	0.838	**< 0.001**
Ahmadi LDL‐C	173.67 (132.21–218.85)	59.38	0.792	**< 0.001**
Puavilai LDL‐C	120.33 (92.13–149.34)	5.03	0.897	**< 0.001**
Vujovic LDL‐C	122.84 (93.43–151.08)	7.25	0.895	**< 0.001**
Chen and Zhang LDL‐C	115.85 (87.27–141.61)	0.80	0.893	0.099
de Cordova LDL‐C	109.36 (78.77–128.91)	‐5.33	0.889	**0.022**
Choi LDL‐C	131.95 (104.77–160.88)	17.64	0.898	**< 0.001**
Martin LDL‐C	119.09 (88.77–145.91)	3.19	0.896	**< 0.001**
Sampson LDL‐C	118.82 (90.84–148.20)	3.50	0.893	**< 0.001**

*Note:* Data presented as median (IQR) depending on the normality. Wilcoxon signed‐rank test *p* values were presented depending on the normality (*p* < 0.05), and values in bold were considered statistically significant.

Abbreviations: IQR, interquartile range; LDL‐C, low‐density lipoprotein cholesterol.

### 3.6. Estimation of LDL‐C in Four Subgroups Based on TG Ranges

In individuals with TG < 150 mg/dL, most formulas including Friedewald, Martin, Sampson, Puavilai, and DeLong showed small median differences (≤ 5 mg/dL) and high correlations (*r* ≥ 0.84). The de Cordova formula slightly underestimated LDL‐C (−11.03 mg/dL), while Ahmadi overestimated it (+41.45 mg/dL) with the lowest correlation (*r* = 0.696). In the TG 150–199 mg/dL group, Martin, Sampson, and Puavilai again maintained high correlations (*r* ≥ 0.89) and small differences (≤ 6 mg/dL), whereas the Ahmadi and Hattori formulas showed large positive biases (+114.30 and +59.80 mg/dL, respectively). The de Cordova and Friedewald equations had minimal differences and nonsignificant *p* values, reflecting good agreement. Among individuals with TG ≥ 200 mg/dL, the Sampson, Martin, and Puavilai formulas continued to exhibit high correlations (*r* ≥ 0.88) and relatively small median differences (< 11 mg/dL), whereas Ahmadi and Hattori formulas overestimated LDL‐C (+174.40 and +83.08 mg/dL, respectively), with Ahmadi showing the lowest correlation (*r* = 0.669). Martin, Sampson, and Puavilai demonstrated the most consistent and reliable performance across all TG strata, particularly in moderate to high TG settings where traditional formulas like Friedewald and Ahmadi showed reduced accuracy (Table 4).

**Table 4:** Median (IQR) values of calculated LDL‐C by 13 formulas for various TG ranges and their correlation with directly measured LDL‐C.

Method	Median (IQR, mg/dL)	Median difference (mg/dL)	Correlation (*r*)	Wilcoxon *p*
TG < 150 mg/dL (*n* = 726)				
Direct LDL‐C	102.57 (78.29–133.19)	—	—	—
Friedewald LDL‐C	110.85 (83.18–139.71)	0.01	0.846	**< 0.001**
DeLong LDL‐C	114.25 (87.40–143.76)	4.28	0.847	**< 0.001**
Rao LDL‐C	119.97 (94.26–150.03)	11.83	0.842	**< 0.001**
Hattori LDL‐C	138.75 (113.25–169.74)	32.01	0.825	**< 0.001**
Anandaraja LDL‐C	116.27 (87.28–148.96)	8.73	0.801	**< 0.001**
Ahmadi LDL‐C	147.73 (118.68–186.20)	41.45	0.696	**< 0.001**
Puavilai LDL‐C	113.69 (86.65–142.96)	3.59	0.847	**< 0.001**
Vujovic LDL‐C	114.97 (88.11–144.73)	5.10	0.847	**< 0.001**
Chen and Zhang LDL‐C	106.30 (82.61–133.88)	‐2.43	0.846	**< 0.001**
de Cordova LDL‐C	96.96 (76.66–119.53)	‐11.03	0.842	**< 0.001**
Choi LDL‐C	124.31 (95.64–153.07)	14.60	0.846	**< 0.001**
Martin LDL‐C	110.15 (83.51–138.70)	0.01	0.846	**0.001**
Sampson LDL‐C	111.94 (84.57–141.90)	2.37	0.847	**< 0.001**
TG 150–199 mg/dL (*n* = 139)				
Direct LDL‐C	109.59 (74.88–142.35)	—	—	—
Friedewald LDL‐C	111.20 (77.43–149.23)	0.00	0.896	**0.048**
DeLong LDL‐C	118.76 (84.36–155.89)	6.98	0.895	**< 0.001**
Rao LDL‐C	140.56 (107.63–182.98)	33.49	0.884	**< 0.001**
Hattori LDL‐C	166.12 (136.08–206.56)	59.80	0.882	**< 0.001**
Anandaraja LDL‐C	114.66 (86.02–148.77)	2.36	0.815	**0.008**
Ahmadi LDL‐C	223.22 (190.89–259.84)	114.30	0.827	**< 0.001**
Puavilai LDL‐C	117.50 (83.25–154.78)	5.84	0.896	**< 0.001**
Vujovic LDL‐C	120.00 (85.60–157.23)	8.32	0.895	**< 0.001**
Chen and Zhang LDL‐C	113.72 (82.70–147.63)	3.76	0.894	**< 0.001**
de Cordova LDL‐C	108.98 (83.25–137.54)	0.10	0.890	0.909
Choi LDL‐C	129.02 (97.46–168.04)	18.35	0.889	**< 0.001**
Martin LDL‐C	116.84 (83.33–152.26)	5.64	0.893	**< 0.001**
Sampson LDL‐C	115.49 (82.25–152.00)	4.16	0.895	**< 0.001**
TG ≥ 200 mg/dL (*n* = 111)				
Direct LDL‐C	113.10 (91.00–158.73)	—	—	—
Friedewald LDL‐C	118.00 (84.41–158.43)	‐0.11	0.879	0.272
DeLong LDL‐C	127.39 (94.57–168.40)	9.40	0.880	**< 0.001**
Rao LDL‐C	190.23 (151.86–235.22)	62.03	0.832	**< 0.001**
Hattori LDL‐C	206.37 (173.74–245.26)	83.08	0.875	**< 0.001**
Anandaraja LDL‐C	118.26 (81.28–165.40)	‐2.26	0.855	0.856
Ahmadi LDL‐C	313.44 (264.51–348.17)	174.40	0.669	**< 0.001**
Puavilai LDL‐C	124.90 (93.23–166.50)	7.84	0.881	**< 0.001**
Vujovic LDL‐C	130.39 (96.18–170.69)	11.48	0.880	**< 0.001**
Chen and Zhang LDL‐C	128.02 (93.96–164.13)	7.56	0.883	**< 0.001**
de Cordova LDL‐C	129.28 (98.46–160.05)	7.12	0.887	**< 0.001**
Choi LDL‐C	141.82 (105.11–181.68)	22.93	0.883	**< 0.001**
Martin LDL‐C	131.12 (96.73–167.28)	10.39	0.891	**< 0.001**
Sampson LDL‐C	122.93 (92.23–161.94)	5.04	0.884	**< 0.001**

*Note:* Data presented as median (IQR) depending on the normality. Wilcoxon signed‐rank test *p* values were presented depending on the normality (*p* < 0.05), and values in bold were considered statistically significant.

Abbreviations: IQR, interquartile range; LDL‐C, low‐density lipoprotein cholesterol; TG, triglyceride.

### 3.7. Estimation of LDL‐C in Three Subgroups Based on TC Ranges

In participants with TC < 200 mg/dL, the Sampson, Martin, Puavilai, and DeLong formulas closely matched direct LDL‐C values (median differences ≤ 5 mg/dL) and exhibited high correlations (*r* ≥ 0.82), while the Ahmadi formula overestimated LDL‐C (+49.98 mg/dL) and showed a weak correlation (*r* = 0.302). In the TC 200–239 mg/dL group, most formulas demonstrated reduced accuracy, with Friedewald, Martin, Sampson, and Puavilai maintaining moderate correlations (*r* = 0.65) and smaller differences (< 5 mg/dL), whereas the Ahmadi and Hattori significantly overestimated LDL‐C (+64.10 and +42.02 mg/dL, respectively) and correlated poorly with the direct method (*r* = 0.217 and *r* = 0.392). In the TC ≥ 240 mg/dL category, Sampson, Choi, and Friedewald formulas demonstrated good performance with strong correlations (*r* ≥ 0.86) and small median differences (≤ 3 mg/dL), while Ahmadi and Hattori again overestimated LDL‐C (+65.23 and +40.56 mg/dL) with low correlations (*r* = 0.320 and *r* = 0.641). Across all TC strata, the Sampson and Martin equations demonstrated consistent reliability and agreement with directly measured LDL‐C, whereas the Ahmadi and Hattori formulas consistently overestimated LDL‐C levels, particularly at lower and higher TC levels, and showed weaker correlations (Table 5).

**Table 5:** Median (IQR) values of calculated LDL‐C by 13 formulas for various TC ranges and their correlation with directly measured LDL‐C.

Method	Median (IQR)	Median difference (mg/dL)	Correlation (*r*)	Wilcoxon *p*
TC < 200 mg/dL (*n* = 604)				
Direct LDL‐C	87.75 (71.76–106.77)	—	—	—
Friedewald LDL‐C	89.34 (69.26–109.45)	0.01	0.827	**0.001**
DeLong LDL‐C	94.35 (75.63–114.12)	4.64	0.827	**< 0.001**
Rao LDL‐C	105.77 (86.87–127.79)	13.74	0.706	**< 0.001**
Hattori LDL‐C	127.04 (108.09–148.14)	35.67	0.644	**< 0.001**
Anandaraja LDL‐C	93.70 (72.85–111.95)	1.21	0.723	**0.001**
Ahmadi LDL‐C	142.71 (114.34–178.89)	49.98	0.302	**< 0.001**
Puavilai LDL‐C	93.47 (74.56–113.32)	3.85	0.827	**< 0.001**
Vujovic LDL‐C	95.20 (76.94–115.00)	5.61	0.826	**< 0.001**
Chen and Zhang LDL‐C	89.47 (73.46–107.79)	0.73	0.822	**0.002**
de Cordova LDL‐C	84.18 (70.35–99.35)	‐3.76	0.781	**< 0.001**
Choi LDL‐C	103.99 (84.90–122.35)	14.17	0.826	**< 0.001**
Martin LDL‐C	91.58 (74.46–110.65)	1.88	0.824	**< 0.001**
Sampson LDL‐C	91.91 (73.62–111.51)	2.30	0.828	**< 0.001**
TC 200–239 mg/dL (*n* = 198)				
Direct LDL‐C	131.24 (113.69–144.77)	—	—	—
Friedewald LDL‐C	137.11 (123.65–149.45)	0.00	0.660	**0.003**
DeLong LDL‐C	141.30 (129.32–154.37)	5.37	0.654	**< 0.001**
Rao LDL‐C	158.12 (139.20–172.17)	19.76	0.424	**< 0.001**
Hattori LDL‐C	177.77 (158.45–194.28)	42.02	0.392	**< 0.001**
Anandaraja LDL‐C	145.94 (137.18–153.97)	14.36	0.332	**< 0.001**
Ahmadi LDL‐C	191.25 (160.51–234.15)	64.10	0.217	**< 0.001**
Puavilai LDL‐C	140.51 (128.12–153.43)	4.50	0.655	**< 0.001**
Vujovic LDL‐C	142.10 (130.51–155.44)	6.48	0.651	**< 0.001**
Chen and Zhang LDL‐C	134.30 (122.60–144.61)	‐2.22	0.632	**0.983**
de Cordova LDL‐C	123.11 (110.50–132.85)	‐12.13	0.529	**< 0.001**
Choi LDL‐C	152.44 (142.46–164.11)	17.75	0.651	**< 0.001**
Martin LDL‐C	137.69 (126.49–149.71)	1.44	0.638	**< 0.001**
Sampson LDL‐C	139.43 (126.63–151.86)	2.93	0.657	**< 0.001**
TC ≥ 240 mg/dL (*n* = 174)				
Direct LDL‐C	177.84 (157.17–205.04)	—	—	—
Friedewald LDL‐C	180.70 (164.03–204.95)	‐0.07	0.864	**0.042**
DeLong LDL‐C	186.09 (169.58–211.97)	5.37	0.858	**< 0.001**
Rao LDL‐C	206.46 (186.74–234.93)	19.69	0.664	**< 0.001**
Hattori LDL‐C	225.22 (205.83–250.77)	40.56	0.641	**< 0.001**
Anandaraja LDL‐C	192.41 (175.26–218.65)	16.93	0.808	**< 0.001**
Ahmadi LDL‐C	245.25 (210.71–307.28)	65.23	0.320	**< 0.001**
Puavilai LDL‐C	185.07 (168.30–210.59)	4.46	0.860	**< 0.001**
Vujovic LDL‐C	187.33 (170.94–213.72)	6.45	0.856	**< 0.001**
Chen and Zhang LDL‐C	173.68 (159.84–196.98)	‐6.87	0.844	**< 0.001**
de Cordova LDL‐C	158.14 (144.68–177.13)	‐23.49	0.785	**< 0.001**
Choi LDL‐C	198.61 (181.90–224.66)	18.81	0.866	**< 0.001**
Martin LDL‐C	180.78 (165.71–206.81)	‐0.23	0.851	**0.025**
Sampson LDL‐C	183.47 (166.59–207.84)	2.89	0.867	**< 0.001**

*Note:* Data presented as median (IQR) depending on the normality. Wilcoxon signed‐rank test *p* values were presented depending on the normality (*p* < 0.05), and values in bold were considered statistically significant.

Abbreviations: IQR, interquartile range; LDL‐C, low‐density lipoprotein cholesterol; TC, total cholesterol.

## 4. Discussion

Estimation of LDL‐C is valuable in assessing cardiovascular risk and therapeutic maneuvers such as dietary modification, initiation of statin therapy, and drug monitoring. Estimation formulas are commonly used in most settings due to the unavailability and cost restrictions of direct measurement of LDL‐C. The Friedewald formula, though widely used, has reduced accuracy in individuals with high TG levels or atypical lipid profiles. Estimating formulas such as those from Sampson, Martin, and Puavilai have been developed to overcome the challenges with direct measurement. Given the variation in lipid variables in various populations, it is important to evaluate the performance of these formulas in different cohorts. This study is aimed at comparing the accuracy of 13 LDL‐C estimating formulas with directly measured LDL‐C in a Ghanaian population.

In this study, the DeLong, Puavilai, Vujovic, Sampson, Choi, and Martin formulas demonstrated the most consistent accuracy with strong correlations and minimal bias across all age groups, TG, and TC levels. However, the correlation coefficients observed in this study are lower than those reported in previous studies, where correlations between calculated LDL‐C and reference methods often exceed 0.95 [35, 36]. This difference may be due to methodological variation, as LDL‐C was measured using a homogeneous enzymatic assay rather than the reference *β*‐quantification method, which may introduce analytical variability and reduce correlation strength. In contrast, the Ahmadi and Hattori formulas frequently overestimated LDL‐C and showed weaker correlations, particularly at extreme TG and TC levels. The Friedewald and de Cordova formulas performed well in individuals with lower TG and TC levels but were less reliable at higher concentrations.

The strong correlations observed for the DeLong, Puavilai, Sampson, and Martin formulas align with findings from other large‐scale validation studies. An analysis of 5,051,467 patients in the United States revealed that the Martin/Hopkins formula achieved the highest concordance with directly measured LDL‐C levels, followed by the Sampson, Puavilai, and DeLong formulas (C. [12]). Similarly, in a large Italian study of 114,774 individuals, the Vujovic, Sampson, and Martin formulas showed the highest concordance with directly measured LDL‐C, with Vujovic having the lowest reclassification rate. The minimal bias observed for Friedewald, Puavilai, Chen and Zhang, de Cordova, Sampson, and Martin formulas suggests that these equations maintain clinically acceptable agreement with direct LDL‐C. However, the significant overestimation by Rao, Hattori, Ahmadi, and Choi formulas and the slight overestimation by Anandaraja and Vujovic highlight the need for careful formula selection based on patient lipid profiles.

In our study, the Friedewald equation (79.9%) outperformed all other equations in agreement with the categorization of the dLDL‐C. A previous study conducted among a large‐scale comparison of 24 LDL‐C estimation equations across two independent cohorts similarly found that Friedewald remained competitive in categorical classification despite its well‐documented analytical shortcomings [35]. Also, Hattori (16.9%) and Ahmadi (17.1%) equations had poor classification performance with less than one in five patients being classified correctly. A previous study by Samuel et al. [12] reported a relatively higher classification accuracy percentage over two times the observed in the present study. This variation could be due to the different reference methods used, while Samuel et al. [12] used *β*‐quantification which is the gold standard and a more accurate technique compared to the direct homogeneous assays used in the present study.

Across all age groups, the Sampson, Martin, Puavilai, and Friedewald formulas consistently showed the smallest median differences and highest correlations with direct LDL‐C. The poor performance of the Ahmadi formula in older adults may reflect algorithmic assumptions that do not hold in aged populations or in those with lipid profiles distinct from the original derivation cohort. A study by Karkhaneh et al. [37] evaluated eight LDL‐C estimation formulas in 2754 Iranian subjects across four age groups. The Ahmadi formula consistently overestimated LDL‐C levels and exhibited poor correlation with direct measurements across all age groups, with particularly low correlation coefficients in older adults [37]. Similarly, the de Cordova formula′s tendency to underestimate LDL‐C in individuals aged 56 and above may be explained by age‐associated shifts in TG‐to‐cholesterol ratios [38]. The de Cordova formula estimates LDL‐C using a fixed multiplier of the difference between TC and HDL‐C, without incorporating TG levels [39]. This omission can lead to underestimation of LDL‐C in older adults due to age‐associated shifts in lipid metabolism [38].

TG and TC strata analyses revealed that Martin, Sampson, Puavilai, and DeLong had high accuracy across all lipid levels, whereas Ahmadi and Hattori formulas significantly overestimated LDL‐C at TC ≥ 240 mg/dL and TG > 150. This disparity likely stems from the latter formulas′ reliance on fixed conversion factors that do not account for the dynamic lipid profile variability [38].

The consistent accuracy and minimal bias of the Sampson, Martin, and Puavilai equations across age, TG, and TC strata support their recommendation for routine clinical use in Ghanaian populations. Given the rising burden of CVD in Ghana and sub‐Saharan Africa, accurate LDL‐C estimation is critical for management. In patients with high TGs or cholesterol, the Hattori and the Ahmadi formula should be avoided to prevent misclassification. Adoption of more accurate equations may improve cardiovascular risk prediction and guide lipid‐lowering therapy more effectively.

This study′s strengths include a large and diverse age range which enhances the generalizability of findings to the Ghanaian population. However, the study has a few limitations. First, the single‐center design and absence of an external validation cohort may restrict broader extrapolation. Additionally, the use of a direct LDL‐C assay as the reference standard is subject to assay variability, which could influence bias estimates. Furthermore, the absence of broader metabolic characterization limits our ability to fully explain population‐specific deviations from previously published equation performance and should be addressed in future validation studies.

Future research should validate these findings in Ghanaian patients with metabolic comorbidities and in nonfasting states, reflecting real‐world clinical scenarios. Exploration of machine learning models tailored to African lipid profiles could further improve LDL‐C estimation accuracy. Cost–benefit analyses are also warranted to assess the feasibility of implementing alternative formulas in routine Ghanaian laboratories.

## 5. Conclusion

The Sampson, Martin, and Puavilai formulas demonstrated the most reliable and consistent performance in estimating LDL‐C compared to direct measurement across diverse age and lipid subgroups in a Ghanaian cohort. These equations should be considered preferred methods for LDL‐C estimation in Ghana, as their adoption will strengthen clinical decision‐making through accurate cardiovascular risk assessment and guidance on lipid‐lowering therapy. While the Friedewald and Chen and Zhang formulas showed acceptable performance in adults aged 18–55 and individuals with TG levels ≥ 200 mg/dL, their overall agreement was lower, highlighting the superior utility of the Sampson, Martin, and Puavilai equations in broader applications.

## Funding

No funding was received for this manuscript.

## Conflicts of Interest

The authors declare no conflicts of interest.

## Data Availability

The datasets used or analyzed during this study are available from the corresponding author upon request.
